# P-1790. Multifaceted Stewardship for Oral Antimicrobial Agents in the Outpatient Setting of a Japanese Tertiary Care Center

**DOI:** 10.1093/ofid/ofae631.1953

**Published:** 2025-01-29

**Authors:** Hiroshi Horiuchi, Yasuaki Tagashira, Asami Takizawa, Yoshiaki Gu

**Affiliations:** National Hospital Organization Yokohama Medical Center, Yokohama City, Kanagawa, Japan; Tokyo Medical and Dental University, Bunkyo-ku, Tokyo, Japan; National Hospital Organization Yokohama Medical Center, Yokohama City, Kanagawa, Japan; Tokyo Medical and Dental University, Bunkyo-ku, Tokyo, Japan

## Abstract

**Background:**

Oral antimicrobials accounted for approximately 90% of all antimicrobial prescriptions. The 2023-2027 Japanese National Action Plan on Antimicrobial Resistance encourages reducing prescriptions for oral broad-spectrum antimicrobials, especially third-generation cephalosporins (3GC), fluoroquinolone (FQ), and macrolide (MC). However, only a few studies have examined antimicrobial stewardship programs (ASP) in the outpatient setting in Japan.

In this study we aim to evaluate the effectiveness of multifaceted ASP for oral antimicrobials in the outpatient setting of a Japanese tertiary care center.

**Methods:**

The present, before-after trial was conducted in the outpatient setting of a Japanese tertiary care center. The pre-intervention period was from October 2021 to September 2023, and the implementation period was from October 2023 to March 2024. The intervention consisted of 1) an infectious disease (ID) physician’s focused, educational lectures for prescribers; 2) local treatment guidelines for common infectious diseases; 3) monthly reports to all physicians on changes in days of therapy (DOT) with 3GC, FQ, and MC per 100 visits; and 4) post-prescription review and feedback from a designated ID physician. Interrupted time-series analysis (ITSA) was used to assess changes in the monthly DOT per 100 visits.

**Results:**

In total, 344,330 and 89,898 patients visited, and 15,324 (4.45%) and 3,845 (4.28%) prescriptions were issued during the pre-intervention and intervention periods, respectively. ITSA revealed an immediate decrease in DOT per 100 visits for 3GC (-1.03; 95% confidence interval [CI]: -1.31 to -0.74; P< 0.001 for intercept) and a decreasing trend in FQ and MC during the intervention period (-0.71 and -0.41; 95% CI: -0.54 to -0.27 and -1.18 to -0.25; P< 0.001 and 0.004, respectively). ITSA also found a decreasing trend in the DOT per 100 visits for all oral antimicrobials (-2.04; 95% CI: -2.88 to -1.20; P< 0.001).

**Conclusion:**

Multifaced ASP was able to reduce the prescription rate of three antimicrobials targeted by the Japanese National Action Plan on Antimicrobial Resistance in the outpatient setting of a community hospital.

**Disclosures:**

**Yoshiaki Gu, MD, PhD, MPH**, MSD K.K.: Grant/Research Support

